# Dual-active antifungal agents containing strobilurin and SDHI-based pharmacophores

**DOI:** 10.1038/s41598-019-47752-x

**Published:** 2019-08-06

**Authors:** Marco Zuccolo, Andrea Kunova, Loana Musso, Fabio Forlani, Andrea Pinto, Giulio Vistoli, Silvia Gervasoni, Paolo Cortesi, Sabrina Dallavalle

**Affiliations:** 1 Department of Food, Environmental and Nutritional Sciences, Università degli Studi di Milano, via Celoria 2, 20133 Milano, Italy; 2 Department of Pharmaceutical Sciences Università degli Studi di Milano, via Mangiagalli 25, 20133 Milano, Italy

**Keywords:** Small molecules, Enzymes, Fungi

## Abstract

Crop disease management often implies repeated application of fungicides. However, the increasing emergence of fungicide-resistant pathogens requires their rotation or combined use. Tank-mix combinations using fungicides with different modes of action are often hard to manage by farmers. An alternative and unexploited strategy are bifunctional fungicides, *i*.*e*. compounds resulting from conjugation of the pharmacophores of fungicides with different mechanisms of action. In this paper we describe a new approach to antifungal treatments based on the synthesis of dual agents, obtained by merging the strobilurin and succinate dehydrogenase inhibitor pharmacophores into a new entity. The compounds were tested against important fungal plant pathogens and showed good inhibition of *Pyricularia oryzae* and *Sclerotinia sclerotiorum* with activity comparable to commercial fungicides. The inhibition of the cytochrome *bc*1 and the succinate dehydrogenase enzyme activity confirmed that the new molecules are endowed with a dual mechanism of action. These results were further supported by molecular modelling which showed that selected compounds form stable complexes with both cytochrome *b* subunit and succinate dehydrogenase enzyme. This work can be considered an important first step towards the development of novel dual-action agents with optimized structure and improved interaction with the targets.

## Introduction

As a constantly growing population is in demand of maximized agricultural yields, a precise pest management system is required, both to reduce the toxicity of pesticide application and minimize environmental hazards. Plant pathogenic fungi are among the major causes of crop losses^1^, and their efficient disease management often implies repeated application of fungicides. As a result, not only the cost of treatments increases, but such usage leads to unfavourable outcomes to the environment, including soil and water pollution. Furthermore, the number of fungal strains resistant to multiple antifungal compounds is dramatically increasing^2^. To overcome the cited drawbacks, a well-established approach is the use of tank-mix combination of molecules with different modes or sites of action. However, such combinations can be less effective than expected due to differential behaviour of each compound, difficulty to achieve optimal concentrations, unpredictable interactions and enhancement of adverse effects^3^.

The design of hybrid bifunctional compounds, *i*.*e*. conjugates resulting from merging the pharmacophores of active molecules with different mechanisms of action, appears to be a promising alternative to the combination approach, since it displays several advantages^4,5^. In particular, synergistic interaction of the two active components able to inhibit simultaneously multiple targets, improved bioactivity and lower risk of resistance are expected^6^. Even though the use of co-formulations or tank-mixes of fungicides with different modes of action is a well established strategy, their conjugation into a single molecule is a relatively underexplored approach. To the best of our knowledge, synthetic studies directed to find dual-action pesticides are very few^7–10^. In regard to antifungal compounds, Cheng *et al*.^10^ described 1,2,4-triazole-1,3-disulfonamides as dual inhibitors of mitochondrial complex II and complex III, whereas other groups reported examples of strobilurins functionalized with a 1,2,3-triazole moiety^9^ or with N-phenylpyrimidin-2-amines^7^.

Strobilurins, or quinone outside inhibitors (QoI), are an outstanding class of fungicides, whose discovery was inspired by a group of natural derivatives of *β*-methoxy acrylic acid, isolated mainly from basidiomycetes^11^. These compounds inhibit mitochondrial respiration by binding to a specific site in the mitochondria, the quinol oxidation (Qo) site (or ubiquinol site) of cytochrome *b* (Cyt *b*; subunit of the Cyt *bc*1 complex) and thereby hamper electron transfer between Cyt *b* and cytochrome *c* (Cyt *c*). This prevents oxidation of reduced nicotinamide adenine dinucleotide (NADH) and synthesis of adenosine triphosphate (ATP), thus leading to the inhibition of the energy production essential for survival^12^. At present, there are numerous synthetic analogues derived from natural strobilurins registered as fungicides in the world market and more are still being developed^13^. Since strobilurins have a single-site mode of action, they are prone to the development of resistance.

Succinate dehydrogenase inhibitors (SDHI) are the fastest growing class of fungicides in terms of new compounds launched into the market^14^. The SDH enzyme (also termed succinate ubiquinone oxidoreductase) is a mitochondrial heterotetramer composed of four nuclear-encoded subunits. In contrast to other dehydrogenases of the tricarboxylic acid (TCA) cycle, the SDH enzyme transfers succinate-derived electrons directly to the ubiquinone pool of the respiratory chain and not to soluble nicotinamide adenine dinucleotide (NAD^+^) intermediates. For this reason, SDH, named also complex II, is considered to be an essential component of the respiratory chain. All crop protection SDHI target the ubiquinone-binding pocket. Upon binding, they physically block the access to the substrate, which consequently prevents further cycling of succinate oxidation. Currently, the “overall” spectrum of SDHI fungicides is extremely broad, being comparable with the QoI spectrum. The most recent SDHI fungicides possess high level of activity against the most important pathogens causing diseases in crops^15^.

In this paper we report the results of our recent efforts to develop new hybrid fungicides, combining the pharmacophoric features of strobilurins and SDH inhibitors. This choice was mainly dictated by the following reasons: both classes of fungicides are already marketed and are characterised by highly specific mode of action on known and validated targets. Moreover, both strobilurins and SDHI are active at the same subcellular level, the mitochondrion. The design, synthesis and biological activity evaluation of the new compounds are described.

## Results and Discussion

The design of dual inhibitors was based on SAR studies reported on both classes of fungicides^14,16^, which allowed to define the key moieties of the new hybrid molecules. A crucial step in the design was the identification of an appropriate combination and outdistancing between the pharmacophoric moieties of strobilurins and SDHI, to drive to compounds characterized by a suitable size and geometrical shape to fit the binding pockets of both enzymes.

The characteristic feature of all QoIs with excellent fungicidal activity is a methyl-(*E*)-*β*-methoxyacrylate pharmacophore, or its bioisosteric equivalent, attached to a phenyl ring with a side chain in an adjacent *ortho*-position. The side chains are usually represented by (hetero)aryloxy or (hetero)aryloxymethyl moieties^17^. On the other side, SDHIs are structurally very diverse, but they all display an essential common feature, which is the amide bond. The “core” moiety, an aromatic or heterocyclic ring, is attached to the carbonyl of the amide bond. The core moiety is essential for binding and *in vivo* potency, and it enters deeply into the active site of SDH. On the amine side of the amide bond, a “linker” is attached. The linker varies but it is usually an alkyl chain, or a substituted or unsubstituted (hetero) aromatic group. The *ortho* position usually carries a large hydrophobic group which is a further component of the molecule^14^.

**Figure 1:**
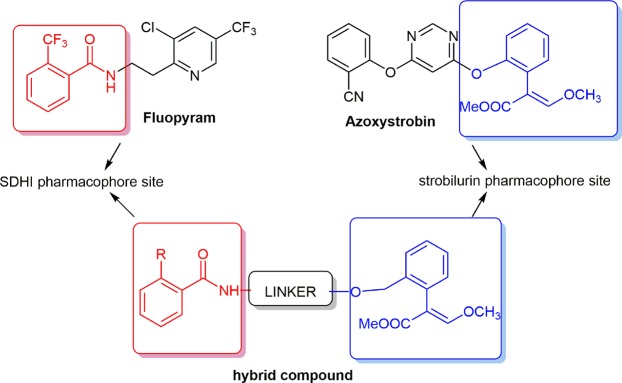
Design of hybrid compounds. The SDHI (red) and the strobilurine (blue) pharmacophoric groups are joined by a linker.

**Figure 2:**
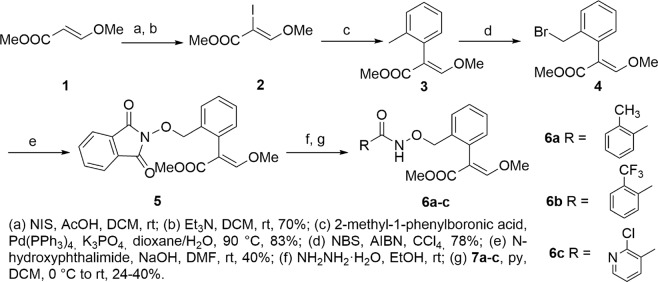
Synthesis of compounds **5** and **6a–c**.

**Figure 3:**
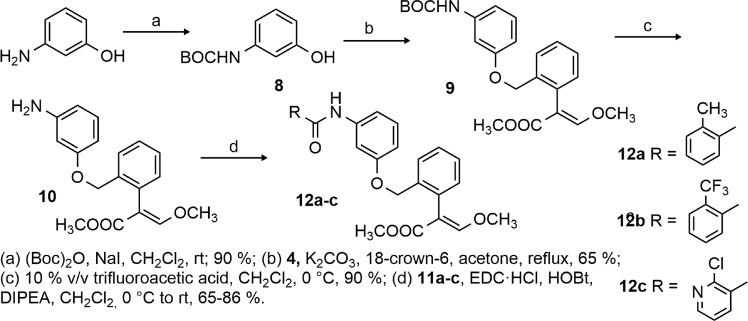
Synthesis of compounds **12a–c**.

Taking these findings into account, we conceived to use as prototypical strobilurin pharmacophore the *β*-methoxyacrylate moiety and to incorporate it into a properly substituted aromatic amide, considered the pharmacophoric group of SDHIs. On the basis of literature evidence, the phenyl ring attached to the *β*-methoxyacrylate should have a spacer linked to the *ortho* position (Fig. 1). Basically, two series of compounds were designed: 1) in the first series the aromatic ring of the SDHI moiety and the aromatic ring linked to the *β*-methoxyacrylate group are connected by a four-atom chain (including the carboxamide group) to maintain the distances between the rings comparable to the distances of the two aromatic rings in azoxystrobin and fluopyram (Fig. 2, compounds **6a–c**); 2) the second series of compounds is characterized by a longer linker containing an aromatic ring (Fig. 3, compounds **12a–c**), capable of giving additional hydrophobic interactions within the binding pockets of both target enzymes.

### Synthesis

The α-iodo-*β*-methoxyacrylate **2** was prepared in good yield from the commercially available *β*-methoxyacrylate **1** following a literature method^18^. Next, compound **2** was coupled with the commercially available 2-methyl-1-phenylboronic acid in a Suzuki-Miyaura reaction in the presence of Pd(PPh_3_)_4_ (4 mol%) and K_3_PO_4_^19^. Despite the good yield obtained, the relatively large amount of catalyst required made this protocol unsuitable for the scale up on gram scale. We observed that the amount of catalyst can be reduced up to 0.5 mol% without significant variations in yield and reaction time. Compound **3** was then converted into the benzyl bromide **4** by radical bromination in the presence of NBS^20^. Compound **4** was coupled with *N*-hydroxyphthalimide to obtain the derivative **5**. The best yield was obtained in the presence of K_2_CO_3_ as a base^21,22^. Finally, **5** was deprotected using hydrazine in absolute ethanol and the unstable free *O*-alkyl hydroxylamine was subjected to acylation without purification. Acylation reactions were performed with acyl chlorides **7a–c** in presence of pyridine as a base^22^ to obtain the final compounds **6a–c** (Fig. 2).

The synthetic strategy to obtain compounds **12a–c** is highlighted in Fig. 3. Protection of the commercially available 3-aminophenol with (Boc)_2_O gave compound **8** which was alkylated with benzyl bromide **4** in presence of K_2_CO_3_ as a base to afford compound **9** in 65% yield. Boc deprotection was easily performed with 10% v/v trifluoroacetic acid in absolute dichloromethane. The reaction afforded the trifluoroacetic salt, which that was converted in the free amine **10** during the work up by treatment with NaHCO_3_ saturated solution. Finally, the amine **10** was coupled with the three acids **11a–c** to obtain the final compounds **12a–c** in good yields. The coupling was performed with EDC·HCl and HOBt as coupling reagents and Hünig’s base.

### Antifungal activity of dual compounds on mycelium growth of fungal plant pathogens

Due to their low solubility in water, the tested molecules were dissolved in acetone^23^. Therefore, two controls were included: MA (malt agar) medium (NTC) and MA supplemented with 1% acetone (ACT).

The antifungal activity of first-series compounds **6a–c** was evaluated in terms of mycelium growth inhibition of 11 fungal strains belonging to 7 different fungal species. Compound **5** was tested as well, considering that it also possessed both strobilurin and SDH inhibitor pharmacophores. The activity of strobilurins on the growth of fungal pathogens is often evaluated in the presence of alternative oxidase (AOX) inhibitors such as salicyl hydroxamic acid (SHAM), which is based on the assumption that while AOX is active *in vitro*, it is not active *in vivo*. However, the use of SHAM *in vitro* is controversial. In fact, it was shown that in some pathogens AOX is active also *in planta*, therefore questioning the use of SHAM *in vitro* to suppress AOX, as it could lead to overestimation of QoI activity^24^. Moreover, inhibitory effect of SHAM on the growth of some fungal pathogens was observed^25,26^, and therefore we evaluated the effect of novel dual compounds and commercial fungicides without the addition of SHAM. The radial growth was measured at 7 DAI, except for fast-growing *S*. *sclerotiorum*, whose growth was evaluated at 3 DAI.

**Figure 4:**
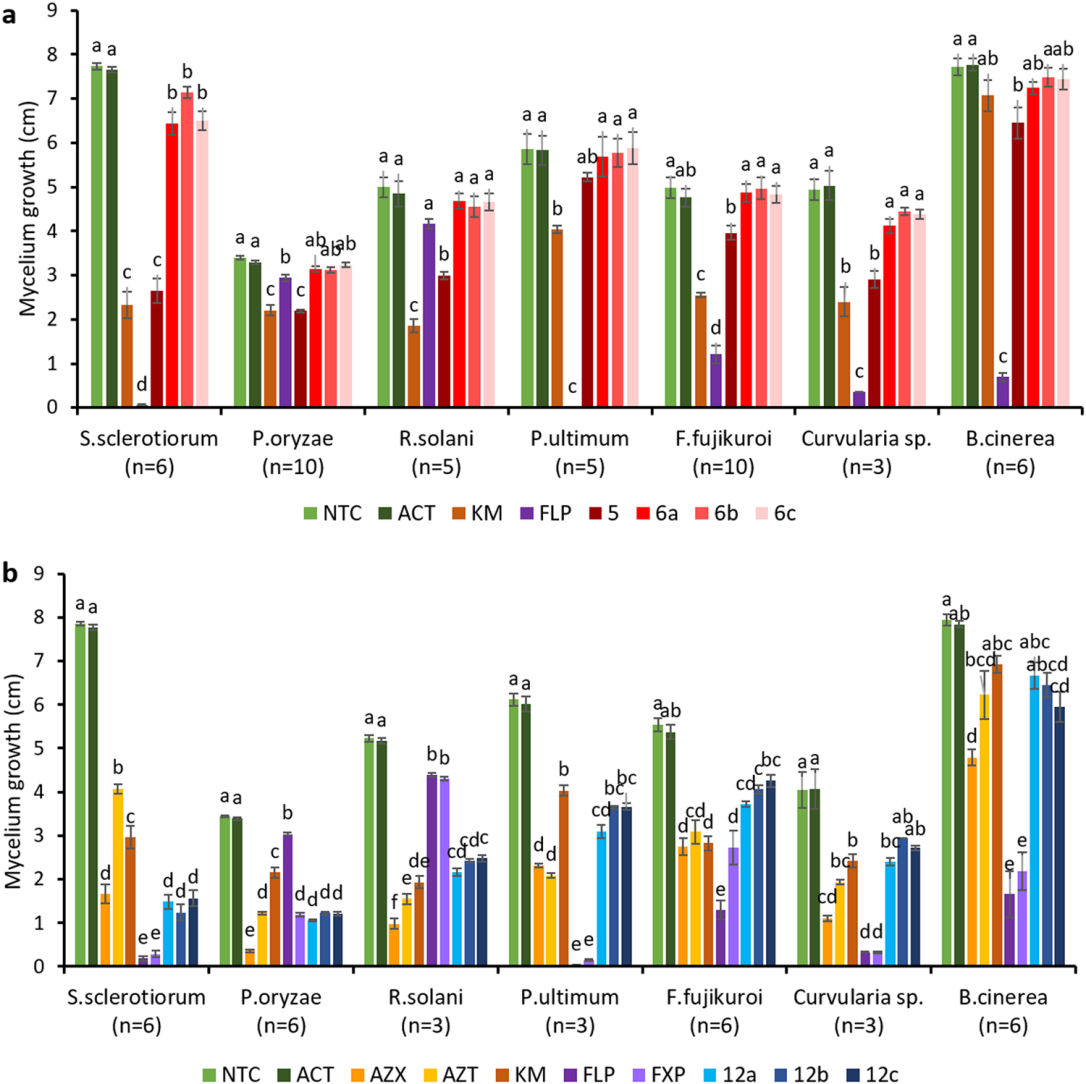
Mycelium growth of seven fungal species on control media (NTC, ACT) or media supplemented with diverse fungicides and dual compounds at the concentration 25 mg/L. (**a**) First-series compounds (**5**, **6a–c**). (**b**) Second-series compounds (**12a–c**). Number of replicates is shown in brackets. NTC – non treated control, ACT – 1% acetone, AZX – azoxystrobin, AZT – technical grade azoxystrobin, KM – kresoxim-methyl, FLP – fluopyram, FXP – fluxapyroxad. The measurements were done at 7 DAI, except for *S*. *sclerotiorum*, which were done at 3 DAI. The error bars represent standard error. The treatments with different letters for each fungal species are significantly different (Tukey HSD, *P* < 0.05).

Among the first-series compounds, out of the four tested molecules, only **5** showed discrete inhibitory activity against selected fungi, while **6a**, **6b** and **6c** showed no or low activity (Fig. 4a). In particular, **5** inhibited the growth of *P*. *oryzae* by ca. 35%, and its activity was comparable with the commercial fungicide kresoxim-methyl (QoI), and even better than that of fluopyram (SDHI). Moreover, **5** inhibited *S*. *sclerotiorum* and *Curvularia* sp. to the same extent of kresoxim-methyl, but less than fluopyram. *P*. *ultimum* and *F*. *fujikuroi* were not inhibited by any of the molecules, whereas they were well controlled by fluopyram.

Similarly, the antifungal activity of compounds **12a–c** was evaluated *in vitro* following the same protocol used for compounds **5** and **6a–c**. The results are highlighted in Fig. 4b. Compounds **12a–c** showed similar activity, with minimal differences against individual pathogens. Moderate inhibitory activity (ca. 50%) was observed against *Curvularia* sp., *P*. *ultimum*, and *R*. *solani*, while *F*. *fujikuroi* and *B*. *cinerea* were only poorly inhibited. The best inhibitory activity for all compounds **12a–c** was observed against *P*. *oryzae* and *S*. *sclerotiorum*. *S*. *sclerotiorum* was inhibited by compounds **12a–c** in a range of ca. 70–80%. The activities were comparable to strobilurins, but inferior to SHDI. Significantly, **12a–c** inhibited the growth of *P*. *oryzae* in a range of 60–70% and their activity was better than that of kresoxim-methyl and fluopyram and comparable to that of fluxapyroxad. Formulated azoxystrobin, which is the only commercial fungicide currently registered in the EU for the control of rice blast, showed the best inhibitory activity against *P*. *oryzae*. However, the technical grade azoxystrobin (AZT) showed activity comparable with the three tested dual molecules **12a–c**. Interestingly, statistical differences were observed between technical grade and commercial azoxystrobin in *S*. *sclerotiorum*, *P*. *oryzae* and *R*. *solani*, but not in other fungi, which confirms that the formulations are specific for particular fungal pathogens and that they significantly influence the performance of the selected fungicide. This also indicates that the activity of compounds **12a–c** can be further increased by proper formulation.

### SDHI and QoI action of the designed compounds

**Figure 5:**
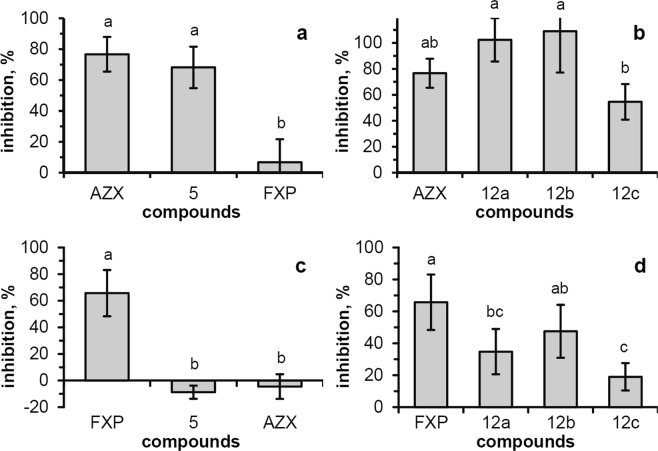
Effect of compounds **5** and **12a–c** on the *P*. *oryzae* A2.5.2 mitochondrial DBH_2_:Cyt *c* reductase and SDH activities. (**a**,**b**) Rate of the Cyt *c*-reduction mediated by the mitochondrial fraction was measured at 30 °C (λ = 550 nm), using decylubiquinol as electron-donor substrate, in the presence of **5**, **12a–c** and reference compounds AZX and FXP (20 µM) to evaluate the percent of inhibition due to the QoI action. (**c**,**d**) Rate of the succinate:2,3-dimethoxy-5-methyl-p-benzoquinone electron transfer mediated by the mitochondrial fraction was measured at 30 °C (λ = 595 nm) using DCPIP in the presence of **5**, **12a–c** and reference compounds AZX and FXP (50 µM – FXP, AZX, **5**; or 80 µM – **12a–c**) to evaluate the percent of inhibition due to the SDHI action. Data represent the mean ± standard deviation of at least three independent trials and different superscript letters indicate statistically significant differences (panels a and c: Tukey HSD, p ≤ 0.01; panels b and d: Tukey HSD, p ≤ 0.05).

To separately assess the action of the strobilurin-like (QoI) and SDHI pharmacophores in the putative dual compounds (compound **5** and compounds **12a–c**), specific sets of experiments were carried out using the mitochondrial preparations of *P*. *oryzae* A2.5.2 in a cell-free system (Fig. 5).

The measurement of the DBH_2_:Cyt *c* reductase activity (*i*.*e*. the Cyt *bc*1 complex activity) was used to evaluate the action of the QoI pharmacophore of compound **5**. To this aim, the reduction rate of Cyt *c* mediated by the mitochondrial fraction of *P*. *oryzae* A2.5.2 was measured, using decylubiquinol (DBH_2_) as electron-donor substrate (Fig. 5a). The Cyt *c*-reduction rate in the presence of the compound **5** (20 μM) was 68.2 ± 13.4% lower than that measured in the control containing DMSO. Interestingly, the inhibition with compound **5** at 100 μM (83.1 ± 10.9%) almost reached the saturation. Azoxystrobin (20 μM), used as positive control in the same assay conditions, showed an inhibition of the Cyt *c*-reduction rate (76.7 ± 11.2%) similar to that of compound **5**. As expected, fluxapyroxad (20 μM), used as negative control, in the same assay conditions showed no clearly detectable inhibition of the Cyt *c*-reduction rate (6.7 ± 15%). Similarly, compound **5** was tested also for the ability to inhibit the SDH activity. For this test, the electron transfer rate mediated by the mitochondrial fraction of *P*. *oryzae* A2.5.2 in the presence of 2,3-dimethoxy-5-methyl-*p*-benzoquinone and succinate, was measured by a colorimetric method using the redox dye 2,6-dichlorophenolindophenol (DCPIP, Fig. 5c). Inhibition of the SDH activity (65.7 ± 17.4%) was observed for fluxapyroxad (50 μM). Conversely, no inhibition was observed for compound **5** and azoxystrobin. The obtained results for compound **5** support the hypothesis that this compound has only a strobilurin-like action.

Inhibition of DBH_2_:Cyt *c* reductase activity was also observed for second-series compounds **12a–c** (Fig. 5b). Compounds **12a**,**b** (20 μM) showed a complete inhibition, while for compound **12c** a lower inhibition (54.6 ± 13.7%) was obtained. Compounds **12a–c** were also tested for the ability to inhibit the SDH activity (Fig. 5d). Indeed, inhibition of the SDH activity was observed for all the tested compounds (80 µM). Out of three tested molecules, **12b** (47.6 ± 16.6%) had an activity comparable with that of FXP (50 μM), while compounds **12a** and **12c** showed lower activity than FXP (50 μM). No inhibition was observed for azoxystrobin.

Thus, according to the overall inhibition data on the target mitochondrial enzyme activities, compounds **12a–c** show the typical target actions associated to both QoI and SDHI pharmacophores.

### Molecular modelling studies

With a view to rationalizing the dual activity, docking simulations were performed to explore the binding mode of the new molecules to both Cyt *b* subunit and succinate dehydrogenase enzyme from *P*. *oryzae*. In both cases, the proteins were modelled by homology modelling techniques and docking results were analyzed by comparison with known ligands.

The available sequence for Cyt *b* from *P*. *oryzae* comprises only a 170 residue-fragment, which is unsuitable for modelling the entire binding pocket. Thus, the Cyt *b* homology model was based on the primary sequence of the Cyt *b* protein from closely related *P*. *grisea* (Uniprot code: Q85KP9). The two sequences are substantially identical in the common region with only two conserved differences out of 170 residues, suggesting a very high identity degree between *P*. *grisea* and *P*. *oryzae*. Therefore, the structure of Cyt *b* from *P*. *grisea* was modelled by using the resolved Cyt *b* structure from *S*. *cerevisiae* in complex with the inhibitor Stigmatellin A (PDB ID: 3CX5) as the template.

The SDH structure is composed of four subunits which have to be modelled together, since the binding site is located at the interface among them. In detail, the homology model was based on three primary sequences from *P*. *oryzae* (i.e. Subunit A: G4NE44; Subunit B: L7JQS7; Subunit C: G4N2P5) while the sequence from *P*. *grisea* was used for subunit D (Q5G5B2), since the corresponding sequence from *P*. *oryzae* is unavailable. Then, the SDH structure was generated based on the resolved structure of the porcine enzyme in complex with N-[(4-tert-butylphenyl)methyl]-2-(trifluoromethyl)benzamide (E23).

**Figure 6:**
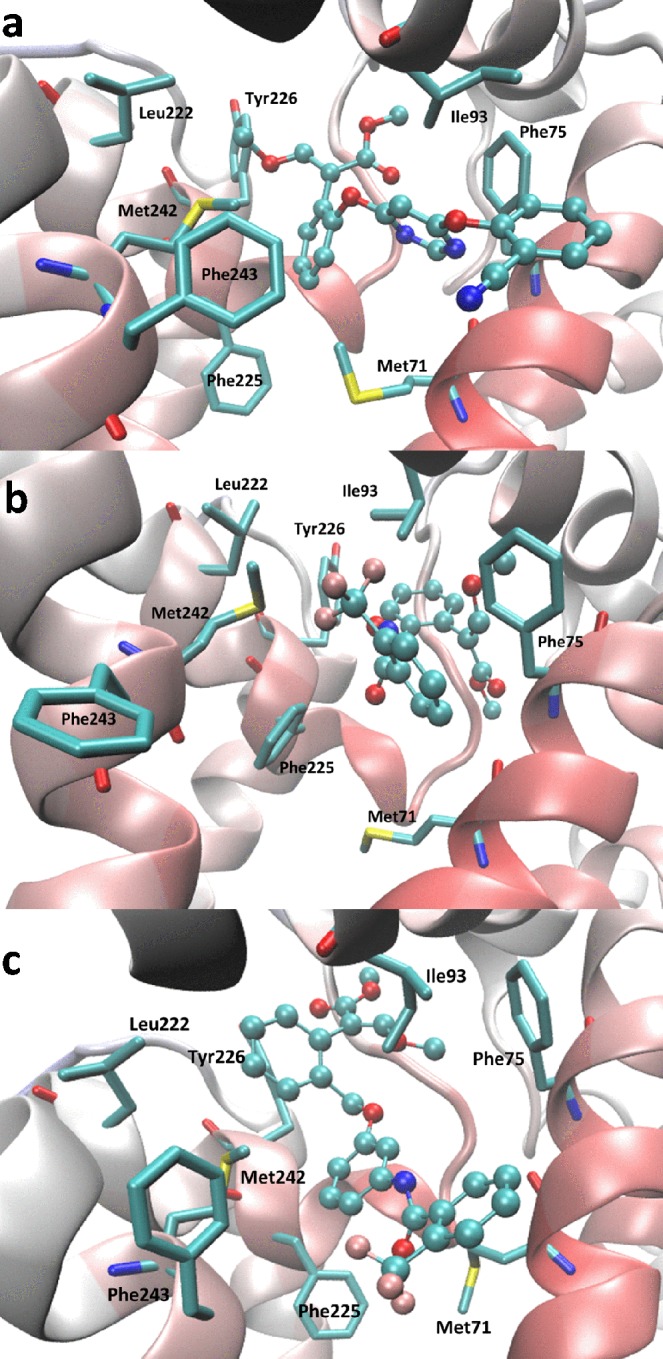
Comparison of the putative poses as computed for (**a**) azoxystrobin, (**b**) **6b** and (**c**) **12b** within the Cyt *b* binding site.

With regard to the docking simulations with Cyt *b*, all tested compounds (**5**, **6a–c** and **12a–c**) showed a binding mode and an interaction pattern similar to that seen for azoxystrobin, which was included in the simulations for easy comparison. Fig. 6 compares the poses as computed for **6b**, **12b** and azoxystrobin, and reveals that all complexes are almost exclusively stabilized by hydrophobic contacts. In detail, the methyl (*E*)- *β*-methoxyacrylate moiety of the ligands is inserted within a highly hydrophobic subpocket, where it interacts with both alkyl (Val92, Leu96, Pro218, Leu222, Leu229) and aryl (Phe75, Tyr226) sidechains. The phenyl ring elicits π–π stacking with Phe225 plus S/π interactions with Met242. The variable portions linked to the phenyl ring are similarly accommodated within an apolar subpocket where they can contact Met71, Ala72, Phe75, Leu76, Ile93 and Phe243. In clear agreement with what was observed for the resolved complex between Bovine cytochrome *bc*1 and azoxystrobin (PDB ID: 1SQB), the computed complexes are completely devoid of polar interactions and there are no surrounding polar residues which might be at least involved in plausible water-bridged H-bonds. On these grounds, the difference in bioactivity seen for the here proposed compounds can be mostly ascribed to the capacity of the variable portion to stabilize π–π contacts with aromatic residues. Accordingly, the good activity of **5** can be explained by considering the enriched stacking interactions that the phthalimide moiety elicits with Phe243^27^ (Figure S1). Similarly, the notable activity of **12a–b** can be explained by considering the reinforced hydrophobic contacts stabilized by these extended derivatives. The reduced inhibitory activity of **12c** can be rationalized considering that the pyridine ring is less effective in stabilizing T-shape π–π stacking, as seen with Phe75 and to minor extent with Phe125.

**Figure 7:**
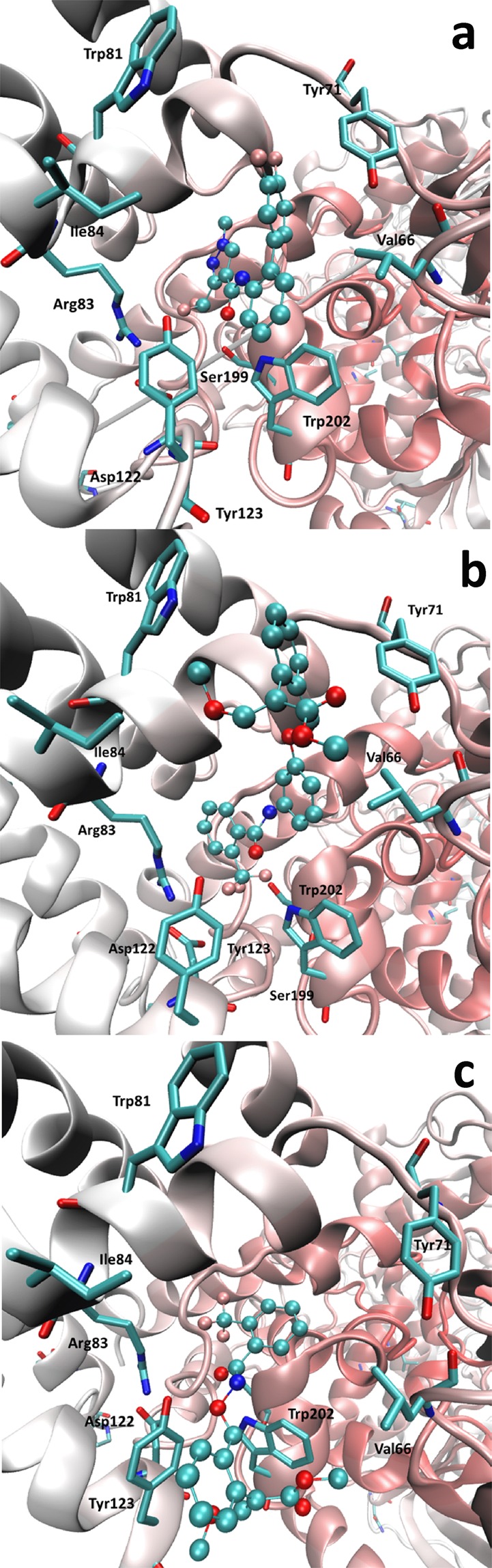
Comparison of the putative poses as computed for (**a**) fluxapyroxad, (**b**) **6b** and (**c**) **12b** within the SDH binding cavity.

Concerning the docking studies involving the SDH enzyme, Fig. 7 compares the computed poses for fluxapyroxad (used here as a reference) and **6b** and **12b** derivatives, revealing some common features between the reference compound and **12b** which can clearly explain its good activity. In detail, both ligands similarly accommodate the carbonyl group, which elicits two key H-bonds with Tyr123 and Trp202, as well as the trifluoromethyl substituent which is engaged in halogen bonds with Asp122, Tyr123 and Ser199. While assuming a slightly different pose, the phenyl ring approaches Arg83 with which it can stabilize charge transfer interactions, similarly to what is observed for the pyrazole ring of fluxapyroxad. Greater differences are detected when comparing the pose of the anilino moieties of the two inhibitors. Due to the marked steric hindrance induced by the (*E*)-*β*-methoxyacrylate moiety group, the *N*-linked phenyl ring of **12b** assumes a shifted pose (compared to fluxapyroxad) by which it is able to insert the phenyl (*E*)- *β*-methoxyacrylate moiety within a suitable and rather external subpocket. Here the phenyl ring approaches Tyr71 and Trp81 and the (*E*)-*β*-methoxyacrylate group is engaged in a H-bond with Tyr71 plus a set of hydrophobic contacts with Leu66, Leu76, Val80 and Ile84. The greater inhibition activity of fluxapyroxad can be ascribed to its capacity to better approach Trp202 with the *N*-linked phenyl ring thus reinforcing the key H-bond between Trp202 and the ligand carbonyl function. In contrast, the inactivity of the compounds of the first series (as here exemplified by the complex with **6b**, Fig. 7b, and compound **5**, Figure S2) can be justified by considering that their phenyl (*E*)-*β*-methoxyacrylate moiety, instead of being accommodated within the mentioned subpocket, assumes a pose comparable with that of the anilino ring of fluxapyroxad. Thus, the (*E*)-*β*-methoxyacrylate detrimentally bumps against Tyr123 and Trp202 and destabilizes their crucial interactions with the inhibitors.

## Conclusion

In this paper we report our first efforts to exploit an innovative approach based on the use of hybrid fungicides. In particular, we combined the pharmacophoric features of two classes of commercially available antifungal compounds: strobilurins and SDH inhibitors. The design of dual inhibitors was based on reported SAR studies on both classes of fungicides, which allowed to define the key moieties of the new molecules. Two series of hybrid compounds, characterized by a variously *ortho*-substituted aromatic carboxamide moiety connected to a *β*–methoxyphenylacrylate, were synthesized and tested to evaluate their inhibitory activity on mycelial growth of diverse fungal pathogens.

Out of the tested molecules belonging to the first series, compound **5** showed the highest inhibitory activity against a series of plant pathogenic fungi. In particular, **5** inhibited the growth of *P*. *oryzae*, one of the most detrimental pathogens worldwide^28^, with activity comparable to commercial fungicide kresoxim-methyl, and even better than that of fluopyram. The compound was tested to assess the inhibition of the Cyt *bc*1 complex and SDH enzyme activities. Inhibition was observed for the Cyt *bc*1 complex enzyme activity, while no inhibitory activity was observed for the SDH enzyme activity. These results indicate that compound **5** is able to express only a strobilurin-like activity, findings which were further confirmed by docking studies. The docking of compound **5** with the computational model of the Cyt *bc*1 complex subunit Cyt *b* showed a binding mode similar to that of the commercial fungicide azoxystrobin, whereas a binding mode different from the standard was observed for **5** in the active site of SDH enzyme. The observed differences were attributed to the steric hindrance of the (*E*)-*β*-methoxy acrylate group.

The second series of compounds (**12a–c**) were designed to increase the distance between the two moieties, which might improve the interaction with both binding pockets. This change led to increased biological activity against all tested fungal pathogens. In particular, good activities were observed against *P*. *oryzae* and *S*. *sclerotiorum*. All the tested compounds showed strong inhibition of the Cyt *c* reduction. More importantly, inhibition of SDH enzyme was observed as well. Therefore, these results strongly indicate that compounds **12a–c** act as true dual target fungicides expressing both the activity of SDH inhibitors and strobilurins.

Molecular modelling studies further confirmed that the selected compounds are capable of forming stable complexes with both enzymes, providing a computational support to our objective to combine the strobilurin and SDHI-like motifs in a single molecule. Further work is required to develop analogues with optimized structure and improved interaction with the selected targets. Moreover, even if this work was focused on *P*. *oryzae* as a model organism to determine the SDHI and QoI action, the approach can be extended to other fungi causing disease on a wide range of economically important crops.

## Materials and Methods

### Chemistry

The detailed synthetic procedures for all the compounds are reported in supplementary material.

### Antifungal activity evaluation

#### Fungal strains

The following fungal and oomycete strains, belonging to a collection maintained in the laboratory of Plant Pathology at DeFENS, University of Milan, Italy, were used in the study: *Botrytis cinerea* BC4A and BC-2F-2016, *Curvularia* sp. CURV3, *Fusarium fujikuroi* FFM2 and FFM4, *Pyricularia oryzae* A2.5.2 and TA102, *Pythium ultimum* FW407 and *Sclerotinia sclerotiorum* SW361 and SSP1. *Rhizoctonia solani* FW408 was obtained from Dr. Andrea Minuto (Centro di Sperimentazione e Assistenza Agricola, Albenga, Italy). The strains were maintained as single-spore isolates on malt-agar medium (MA: 20 g/L malt extract, 15 g/L agar) at 4 °C.

#### Fungicides

The following fungicides were used in the study: azoxystrobin (AZX, Amistar SC – suspension concentrate, 22.9% ai., Syngenta Crop Protection), technical grade azoxystrobin (AZT, Sigma-Aldrich), kresoxim-methyl (KM, Sovran WG – wettable granules, 50% ai., BASF Italia, S.p.A.), fluopyram (FLP, Luna Privilege SC, 50% ai., Bayer CropScience, S.r.l) and fluxapyroxad (FXP, Sercadis EC – emulsifiable concentrate, 30% ai., BASF Italia, S.p.A.).

#### Inhibition of mycelium growth on media supplemented with fungicides

The inhibitory activity of the first-series molecules **5** and **6a–c** and commercial fungicides KM and FLP on mycelium growth of different fungal species was evaluated in a pilot study. A mycelium plug (0.5 cm in diameter) obtained from actively growing fungal colonies of *B*. *cinerea* BC4A and BC-2F-2016, *Curvularia* sp. CURV3, *F*. *fujikuroi* FFM2 and FFM4, *P*. *oryzae* A2.5.2 and TA102, *P*. *ultimum* FW407, *R*. *solani* FW408 and *S*. *sclerotiorum* SW361 and SSP1 was transferred to MA medium plates supplemented or not with commercial fungicides (KM, FLP) and compounds **5**, **6a**, **6b** and **6c** at the concentration 25 mg/L ai. Because the tested dual molecules had low solubility in water, they were dissolved in acetone. Therefore, two controls were included: MA medium (NTC, not treated control), and MA medium supplemented with acetone at the final concentration 1% v/v (ACT). The plates were incubated at 24 °C in the dark. The mycelium growth was measured 3–7 days after inoculation (DAI) and the inhibition of mycelium growth (%) was calculated by comparing the mycelium growth on control and fungicide-supplemented plates.

The activity of the second-series dual molecules **12a–c** was evaluated against the same fungal pathogens as described above. Again, two controls were included: NTC and ACT. The commercial fungicides were AZX, KM, FLP and FXP at the concentration 25 mg/L ai. Moreover, technical grade azoxystrobin (AZT, 25 mg/L ai) was included for comparison. The plates were incubated at 24 °C in the dark and the mycelium growth was measured at 7 DAI.

All experiments were performed in at least three replicates. For statistical analysis, the mycelium growth data of the strains belonging to the same fungal species were pooled together and were submitted to ANOVA followed by a Tukey post hoc test for multiple comparison (*P* < 0.05), using the TukeyC package and R software, version R3.0.3^29,30^.

### SDHI and QoI activity of the designed compounds

#### Preparation of *P*. *oryzae* mitochondrial fraction

The mitochondrial fraction was prepared according to Ye and coworkers^31^ with some modifications. Mycelium liquid culture (200 mL, 20 g/L malt extract, in 1 L flask) was inoculated with 10 mycelium-MA plugs taken from the edge of actively-growing *P*. *oryzae* (A2.5.2) colony and incubated at 25 °C in an orbital shaker (125 rpm) in the dark for 6 days. Mycelium was cooled on ice and harvested by vacuum filtration through Miracloth (Calbiochem^®^, La Jolla, CA, USA). During vacuum filtration residual culture medium was removed by washing 3-times with 30 mL of ice pre-cooled water. The collected well-drained mycelium was chopped in small pieces by scalpel and thoroughly powdered in mortar and pestle in the presence of liquid nitrogen. The mycelium powder (∼ 6 g) was suspended in 10 volumes of ice-cooled mitochondrial buffer (0.5 M sucrose, 10 mM KH_2_PO_4_, 10 mM KCl, 10 mM MgCl_2_, 0.2 mM EDTA, pH 7.2), and gently mixed for 60 min by magnetic stirrer at 4 °C. The suspension was centrifuged two-times for 10 min at 4 °C, 5500 rpm (rotor JA20, Beckman). The cleared supernatant was further centrifuged for 20 min at 4 °C, 11250 rpm (rotor JA20, Beckman). The collected pellet (~ 33 mg) was suspended in mitochondrial buffer (2 mL), divided in 2 Eppendorf tubes and centrifuged again for 20 min at 4 °C, 10000 rpm (rotor F45-24-11, Eppendorf). Each pellet was resuspended in 0.2 mL of the same buffer, and stored at −80 °C until use.

### Enzyme assays

The strobilurin-like (QoI) action was evaluated measuring the decylubiquinol:Cyt *c* reductase activity (*i*.*e*. the Cyt *bc*1 complex activity; EC 1.10.2.2) of the mitochondrial fraction in the presence of the synthesized molecules, using the method described by Zhu *et al*.^7^ with some modifications. The quinol enzyme substrate DBH_2_ was prepared using a NaBH_4_-driven reduction procedure of decylubiquinone^32^. At the end of the procedure, the residue containing DBH_2_ was vacuum-dried using a Savant SpeedVac concentrator and was dissolved in 10 mM HCl-ethanol degassed under nitrogen flow and stored at −80 °C as 0.3 M DBH_2_ aliquots. The enzyme assay was carried out at 30 °C using a spectrophotometer (Lambda 2, Perkin Elmer, USA) equipped with a Peltier system (PTP-6, Perkin Elmer, USA) temperature controller, in 0.5 mL total volume containing the enzyme assay buffer (50 mM KH_2_PO_4_, 3 mM NaN_3_ and 0.01% lauryl maltoside; pH 7.4; extensively degassed under vacuum) with the sequential addition of 20–100 µM tested molecule (25 µL from stocks, freshly prepared in DMSO) or DMSO (25 µL), 40 µM equine heart Cyt *c* (C2506, Sigma Aldrich; 40 µL from ice-cold 0.5 mM stock daily prepared in the enzyme assay buffer), 0.12 mM DBH_2_ (10 µL from 6 mM stock, freshly prepared in ice-cold 10 mM HCl-ethanol degassed under nitrogen flow) and 15 µL of mitochondrial fraction. The Cyt *c* reduction rate was monitored at 550 nm using the first 6-sec time window and was subtracted from the non-enzymatic Cyt *c* reduction rate measured during a 30-sec time interval before the addition of the mitochondrial fraction.

The SDHI action was evaluated measuring the succinate:quinone oxidoreductase (SQR) activity (*i*.*e*. the succinate dehydrogenase activity; EC 1.3.5.1) of the mitochondrial fraction in the presence of the synthesized molecules using a method based on the use of the redox dye, 2,6-dichlorophenolindophenol (DCPIP), and described by Ye *et al*.^29^, with some modifications. The enzyme assay was carried out at 30 °C in 96-well microplates (pre-equilibrated at 30 °C) in 0.2 mL total volume containing 5 µL of mitochondrial fraction (freshly pre-activated by a 30 min incubation, at 30 °C, in the presence of 10 mM sodium succinate), the enzyme assay buffer (50 mM NaH_2_PO_4_, 250 mM sucrose, 3 mM NaN_3_; pH 7.2), 50–80 µM tested molecule (10 µL from stocks, freshly prepared in DMSO) or DMSO (10 µL), 0.20 mM 2,3-dimethoxy-5-methyl-*p*-benzoquinone (10 µL from freshly prepared stock in the enzyme assay buffer; D9150, Sigma Aldrich), 0.14 mM DCPIP (10 µL from freshly prepared stock thoroughly dissolved in the enzyme assay buffer; D1878, Sigma Aldrich). The enzyme reaction was started by the addition of 10 mM sodium succinate (10 µL from freshly prepared 0.2 M stock in the enzyme assay buffer) and was monitored at 595 nm at different fixed times using a fixed wavelength microplate reader set. To calculate the SQR reaction rate, the absorbance decrease due to the DCPIP reduction during the 2–60 min interval time was considered.

In both enzyme assays, each enzyme rate in the presence of the tested molecule (rate_molecule_) was compared to that achieved in the presence of DMSO (rate_DMSO_), in order to calculate the percent of inhibition (I %) as follows:

$${\rm{I}} \% =\frac{{{\rm{rate}}}_{{\rm{molecule}}}-{{\rm{rate}}}_{{\rm{DMSO}}}}{{{\rm{rate}}}_{{\rm{DMSO}}}}\times 100$$

ANOVA followed by *post*-*hoc* Tukey’s HSD test for multiple comparison was used to determine the significance of the data.

### Molecular modelling

The Cyt *b* homology model was based on the primary sequence of the Cyt *b* protein from *P*. *grisea* (Uniprot code: Q85KP9). The structure of Cyt *b* from *P*. *grisea* was modelled by using the resolved Cyt *b* structure from *S*. *cerevisiae* in complex with the inhibitor Stigmatellin A (PDB Id: 3CX5) as the template. Even though the resolved structure of the bovine Cyt *b* protein in complex with azoxystrobin was also available, the study involved the Cyt *b* structure from *S*. *cerevisiae* since the latter shows a higher sequence identity with the Cyt *b* protein from *P*. *grisea* (60% vs. 53% with *S*. *cerevisiae* and bovine cytochrome, respectively).

The SDH structure is composed of four subunits, which have to be modelled together since the binding site is located at the interface among them. In detail, the homology model was based on three primary sequences from *P*. *oryzae* (i.e. Subunit A: G4NE44; Subunit B: L7JQS7; Subunit C: G4N2P5) while the sequence from *P*. *grisea* was used for subunit D (Q5G5B2) since the corresponding sequence from *P*. *oryzae* is unavailable. Then, the SDH structure was generated based on the resolved structure of the porcine enzyme in complex with *N*-[(4-tert-butylphenyl)methyl]-2-(trifluoromethyl)benzamide (E23).

In both cases, the sequence alignment was performed by NIH Cobalt^17^ while the homology models were generated by Modeller9.16^33^. Among the proposed models, the best ones were selected based on DOPE and GA341 scores and optimized by keeping fixed the backbone atoms to preserve the predicted folding. Stigmatellin A and E23 were accommodated within the selected modelled binding sites by superimposing the key residues with those of the utilized templates and the complexes were minimized by keeping fixed all atoms outside a 10 Å radius sphere around the bound ligands. The optimized complexes revealed a set of stabilizing interactions very similar to those seen in the resolved structures, a finding which emphasizes the reliability of the generated homology models.

The conformational profile of the considered ligands was explored by Monte Carlo procedures as described elsewhere^34^.

The docking simulations were performed by PLANTS focusing the search on an 8.0 radius sphere around the bound ligands^35^. For each ligand, 10 poses were generated and scored by the ChemPLP score with a speed equal to 1. Along with the computed primary score, the best complexes were also selected based on the interaction similarity with the complexes obtained with Stigmatellin A and E23. The complexes were finally minimized by keeping fixed all atoms outside a 10 Å radius sphere around the bound ligand.

## Supplementary information

Supporting Information

## Data Availability

The datasets generated during the current study are available from the corresponding author on reasonable request.
