# Supplementary material for: Dual-active antifungal agents containing strobilurin and SDHI-based pharmacophores
Source: Sci Rep. 2019 Aug 6;9:11377. doi: 10.1038/s41598-019-47752-x (PMC6684525; doi:10.1038/s41598-019-47752-x)
Supplement: Supplementary file 1 — Supporting Information [file 41598_2019_47752_MOESM1_ESM.pdf]

Dual-active antifungal agents containing strobilurin and SDHI-based pharmacophores

Marco Zuccolo[1,+], Andrea Kunova[1,+], Loana Musso[1], Fabio Forlani[1], Andrea Pinto[1], Giulio Vistoli[2], Silvia Gervasoni[2], Paolo Cortesi[1], Sabrina Dallavalle[1*]

[1] Department of Food, Environmental and Nutritional Sciences, Università degli Studi di Milano, via Celoria 2, 20133 Milano, Italy
[2] Department of Pharmaceutical Sciences Università degli Studi di Milano, via Mangiagalli 25, 20133 Milano, Italy
[*] sabrina.dallavalle@unimi.it

SUPPORTING INFORMATION

Table of contents

1 Chemistry S2
2 Synthesis S2
3 References S6

Figures

Figure S1 S7
Figure S2 S7

1 Chemistry

All reagents and solvents were reagent grade or were purified by standard methods before use. Melting points were determined in open capillaries on an SMP3 apparatus and are uncorrected. ^{1}H NMR spectra were recorded on 300 MHz and 600 MHz spectrometers; TMS was used as an internal standard. ^{13}C NMR spectra were recorded on 300 MHz and 600 MHz spectrometers. Microanalyses (C, H, N) of new compounds were within ±0.4% of theoretical values. Solvents were routinely distilled prior to use; anhydrous THF and Et_2O were obtained by distillation from sodium benzophenone ketyl; anhydrous CH_2Cl_2 was obtained by distillation from phosphorus pentoxide. All reactions requiring anhydrous conditions were performed under a positive nitrogen flow and all glassware was oven-dried and/or flame-dried. Isolation and purification of the compounds were performed by flash column chromatography on silica gel 60 (230–400 mesh). Analytical TLC was conducted on TLC plates (silica gel 60 F254, aluminium foil). Compounds on TLC plates were detected under UV light at 254 and 365 nm or were revealed by spraying with 10% phosphomolybdic acid (PMA) in EtOH.
Acyl chlorides **7a-c** were prepared as reported in the literature[1–3].
Compound **11a-c** are commercially available.

2 Synthesis

Methyl (Z)-2-Iodo-3-methoxypropenoate **2**.
To a solution of methyl (*E*)-3-methoxypropenoate **1** (3.48 g, 30.0 mmol, 1.0 eq.) in DCM (60 mL), N-iodosuccinimide (8.10 g, 36.0 mmol, 1.2 eq) and glacial acetic acid (4.5 mL, 60 mmol, 2.0 eq) were added at room temperature and the resulting solution was stirred at the same temperature for 24 h. Triethylamine (12.6 mL, 90.0 mmol, 3.0 eq) was added, and the solution was stirred for 12 h at room temperature. Water (90 mL) was added, the layers were separated, and the aqueous layer was further extracted with Et_2O (3 × 60 mL). The combined organic extracts were washed with saturated aqueous solution of $Na_2S_2O_3$ (3 × 60 mL), saturated aqueous solution of $NaHCO_3$ (3 × 60 mL), and water (3 × 60 mL). The organic layer was dried with Na_2SO_4 and the solvent was removed at reduced pressure. The oily residue was purified by flash chromatography (SiO_2, 5:1 petroleum ether/ethyl acetate) to obtain **2** (5.09 g, 70%) as a white solid: mp 50-52 °C; ^{1}H NMR ($CDCl_3$, 300 MHz) δ 7.68 (1H, s), 4.01 (3H, s), 3.81 (3H, s) ^{13}C NMR (75 MHz, $CDCl_3$) δ 52.8, 62.0, 64.8, 164.2, 164.3.

Methyl (E)-2-(2-methylphenyl)-3-methoxyacrylate **3**.
To a solution of methyl (*Z*)-2-Iodo-3-methoxypropenoate **2** (2.50 g, 10.3 mmol, 1.0 eq.) in a 5:1 dioxane/water mixture (52 mL), 2-methyl-1-phenylboronic acid (1.68 g, 12.4 mmol, 1.2 eq.) and K_3PO_4 (6.58 g, 31.0 mmol, 3.0 eq.) were added at room temperature and the resulting solution was degassed with nitrogen over 20 min. Then $Pd(PPh_3)_4$ (59.7 mg, 0.5 mol%) was added, and the reaction mixture was stirred for 6 h at 90 °C in the dark. After cooling to room temperature, the mixture was diluted with ethyl acetate (50 mL) and the organic layer was washed with water (1 × 50 mL) and saturated aqueous solution of NaCl (1 × 50 mL). The organic layer was dried with Na_2SO_4 and the solvent was removed at reduced pressure. The dark oily residue was purified by flash chromatography (SiO_2, 8:1 petroleum ether/ethyl acetate) to afford **3** (mg, 83%) as a colourless oil. Spectroscopic data matched with those reported in the literature[4].

Methyl (E)-2-(2-(bromomethyl) phenyl)-3-methoxyacrylate **4**.
To a solution of methyl (*E*)-2-(2-methylphenyl)-3-methoxyacrylate **3** (2.09 g, 10.1 mmol, 1.0 eq.) in anhydrous carbon tetrachloride (50 mL), N-bromosuccinimide (NBS) (1.98 g, 11.1 mmol, 1.1 eq.) and 2,2'-azo-bisisobutyronitrile (AIBN) (332.2 mg, 2.02 mmol, 0.2 eq.) were added and the resulting suspension

was stirred at reflux in the dark for 6 h. After cooling to room temperature, the mixture was filtered to remove insoluble succinimide and the solid residue was washed with carbon tetrachloride. The solvent was removed at reduced pressure and the oily residue was purified by flash chromatography (SiO_2, 8:1 petroleum ether/ethyl acetate) to afford **4** (2.25 g, 78%) as a white solid: mp 64-65 °C. Spectroscopic data matched with those reported in the literature[4].

2-[2-(1,3-Dioxo-1,3-dihydro-isoindol-2-yloxymethyl)-phenyl]-3-methoxy-acrylic acid methyl ester **5**.
To a solution of N-hydroxyphthalimide (630 mg, 3.86 mmol, 1.1 eq.) in anhydrous *N*,*N*-dimethylformamide (35 mL), K_2CO_3 (728 mg, 5.26 mmol, 1.5 eq.) was added and the resulting red suspension was stirred at room temperature for 20 min. After that, methyl (*E*)-2-(2-(bromomethyl) phenyl)-3-methoxyacrylate **4** (1000 mg, 3.50 mmol, 1.0 eq.) was added and the reaction mixture was stirred at 40 °C overnight. The reaction mixture was poured in cold water and stirred for 1 h. The obtained solid was collected by *in-vacuo* filtration and washed with water. The solid was dissolved in DCM (20 mL) and washed once with water (20 mL). The organic layer was dried with Na_2SO_4 and the solvent was removed at reduced pressure. The residue was recrystallized from 1:4 CH_2Cl_2/hexane to afford **5** (568 mg, 40%) as a white solid: mp 161-163 °C; ^1H NMR ($CDCl_3$, 300 MHz) δ 7.88-7.67 (5H, m), 7.63 (1H, s), 7.47-7.31 (2H, m), 7.20-7.09 (1H, m), 5.12 (2H, s), 3.75 (3H, s), 3.61 (3H, s); ^{13}C NMR ($CDCl_3$, 75 MHz) δ 167.9, 163.5, 160.7, 134.4, 133.3, 133.2, 131.1, 130.6, 129.2, 129.0, 128.1, 123.4, 77.5, 62.0, 51.7. Anal. Calcd. for $C_{20}H_{17}NO_6$: C, 65.39; H, 4.66; N, 3.81. Found: C, 65.60; H, 4.67; N, 3.80.

General procedure for the synthesis of **6a**-**c**.
To a solution of compound **5** (100 mg, 0.27mmol, 1.0 eq.) in methanol (2 mL), hydrazine hydrate (15 µL, 0.30 mmol, 1.1 eq.) was added dropwise and the resulting solution was stirred at room temperature for 4 h. After that, the solution was cooled to 0 °C and the white solid was removed by *in-vacuo* filtration. The methanolic solution was evaporated to dryness and the residue was suspended with few mL of Et_2O. The white solid was removed by *in-vacuo* filtration, the ethereal solution was dried with anhydrous $CaCl_2$ and used for the next reaction without further purification.
The ethereal solution was dried until dryness with a stream of nitrogen in a two necked round bottom flask. The residue was dissolved in anhydrous CH_2Cl_2 (2 mL) and the resulting solution was cooled to 0 °C. A solution of the suitable acyl chloride **7a**-**c** (0.32 mmol, 1.2 eq.) in anhydrous CH_2Cl_2 (2 mL) and anhydrous pyridine (66 µL, 0.81 mmol, 3.0 eq.) were added dropwise at the same temperature and the resulting solution was stirred at room temperature overnight. The reaction mixture was diluted with CH_2Cl_2 (6 mL) and washed with $NaHCO_3$ saturated aqueous solution (10 mL × 1) and NaCl saturated aqueous solution (10 mL × 1). The organic phase was dried with Na_2SO_4, the solvent was removed at reduced pressure, and the residue was purified by flash chromatography (SiO_2, 2:1 to 1:1 hexane/ethyl acetate).

3-Methoxy-2-[2-(2-methyl-benzoylaminooxymethyl)-phenyl]-acrylic acid methyl ester **6a**.
Prepared according to the general procedure (29 mg, 30%) as a white waxy solid.
^1H NMR ($CDCl_3$, 300 MHz) δ 8.46 (1H, s), 7.54 (1H, s), 7.49-7.42 (1H, m), 7.42-7.30 (2H, m), 7.30-7.11 (9H, m), 5.00 (2H, s), 3.77 (3H, s), 3.66 (3H, s), 2.38 (3H, s); ^{13}C NMR ($CDCl_3$, 75 MHz) δ 171.6, 168.5, 159.7, 137.0, 136.9, 134.8, 134.1, 133.5, 133.1, 130.7, 130.4, 129.0, 128.8, 128.3, 127.3, 126.0, 125.9, 125.7, 110.8, 76.4, 62.0, 51.8, 19.0. Anal. Calcd. for $C_{20}H_{21}NO_5$: C, 67.59; H, 5.96; N, 3.94. Found: C, 67.33; H, 5.98; N, 3.95.

3-Methoxy-2-[2-(2-trifluoromethyl-benzoylaminooxymethyl)-phenyl]-acrylic acid methyl ester **6b.**
Prepared according to the general procedure (44 mg, 40 %) as a white solid. mp 137-139 °C. ^1H NMR ($CDCl_3$, 300 MHz) δ 8.63 (1H, s), 7.73-7.59 (1H, m), 7.54 (1H, s), 7.54-7.41 (4H, m), 7.41-7.30 (4H, m), 7.30-7.23 (2H, m), 7.19-7.12 (1H, m) 4.99 (2H, s), 3.79 (3H, s), 3.67 (3H, s); ^{13}C NMR ($CDCl_3$, 75 MHz) δ 168.8,

164.9, 159.8, 134.6, 133.5, 132.5, 132.0, 131.4, 130.7, 130.4, 129.1, 129.0, 128.2, 126.6, 110.6, 76.6, 62.1, 51.8. Anal. Calcd. for $C_{20}H_{18}F_3NO_5$: C, 58.68; H, 4.43; N, 3.42. Found: C, 58.81; H, 4.42; N, 3.42.

2-{2-[(2-Chloro-pyridine-3-carbonyl)-aminooxymethyl]-phenyl}-3-methoxy-acrylic acid methyl ester **6c**. Prepared according to the general procedure (10.2 mg, 24 %) as a light yellow solid. mp 144-147 °C. ^{1}H NMR ($CDCl_3$, 300 MHz) δ 9.12 (1H, s), 8.45-8.36 (1H, m), 7.81-7.71 (1H, m), 7.55 (1H, s), 7.49-7.41 (2H, m), 7.42-7.29 (4H, m), 7.26-7.20 (2H, m), 7.19-7.12 (1H, m), 5.01 (2H, s), 3.80 (3H, s), 3.68 (3H, s); ^{13}C NMR ($CDCl_3$, 75 MHz) δ 168.7, 159.9, 151.2, 150.1, 139.3, 137.0, 134.4, 133.6, 131.6, 130.8, 129.2, 128.3, 122.8, 122.5, 110.6, 76.6, 62.1, 51.9. Anal. Calcd. for $C_{18}H_{17}ClN_2O_5$: C, 57.38; H, 4.55; N, 7.43. Found: C, 57.16; H, 4.55; N, 7.41.

1,1-Dimethylethyl N-(3-hydroxyphenyl)carbamate **8**.
To a solution of 3-aminophenol (500 mg, 4.58 mmol) and NaI (686.7 mg, 4.58 mmol) in abs. THF (10 mL), $(Boc)_2O$ (1.26 mL, 5.50 mmol) was added dropwise at 0 °C and the reaction mixture was stirred at room temperature for 1 h. The reaction mixture was diluted with ethyl acetate (20 mL), washed with a saturated solution of $Na_2S_2O_3$ (1 × 20 mL) and with a saturated solution of $NaHCO_3$ (1 × 20 mL). The organic layer was dried with Na_2SO_4 and the solvent was removed at reduced pressure. The oily residue was triturated with a mixture of 10 % ethyl acetate in hexane to obtain **8** (862 mg, 90 %) of the title compound as a white solid: mp 130-132 °C. Spectroscopic data matched with those reported in the literature[5].

Methyl (E)-2-[2-(3-tert-butoxycarbonylamino-phenoxymethyl)-phenyl-3-methoxy-acrylate **9.**
Compound **8** (387 mg, 1.85 mmol) was added at room temperature to a suspension of anhydrous K_2CO_3 (302 mg, 2.18 mmol) in abs. acetone (11.6 mL) and the resulting mixture was stirred at reflux for 1 h. After that, 18-crown-6 (929 µL, 4.36 mmol) and benzyl bromide **4** (480 mg, 1.68 mmol) were added and the reaction mixture was stirred at reflux for 9 h. The suspended K_2CO_3 was removed by vacuum filtration and the filtrate was removed at reduced pressure. The residue was purified by flash chromatography (SiO_2, 3:1 petroleum ether/ethyl acetate) to obtain compound **9** (452 mg, 65 %) as a white waxy solid.^{1}H NMR (300 MHz, $CDCl_3$): δ = 7.59 (s, 1H), 7.57-7.50 (m, 1H), 7.37-7.27 (m, 2H), 7.20-7.08 (m, 2H), 7.00-6.94 (m, 1H), 6.94-6.90 (m, 1H), 6.57 (ddd, 1H, *J* = 8.2, 2.5, 0.9 Hz), 6.50 (bs, 1H), 4.94 (s, 2H), 3.83 (s, 3H), 3.72 (s, 3H), 1.50 (s, 9H). ^{13}C NMR (75 MHz, $CDCl_3$): δ = 168.1, 160.3, 159.6, 152.7, 139.7, 136.3, 131.3, 131.1, 129.8, 128.2, 127.7, 127.6, 111.0, 110.2, 109.7, 105.3, 80.5, 68.1, 62.1, 51.8, 28.5.

Methyl (E)-2-[2-(3-aminophenoxymethyl)-phenyl]-3-methoxyacrylate **10.**
To a solution of **9** (202 mg, 0.49 mmol) in abs. CH_2Cl_2 (4.9 mL), trifluoroacetic acid (0.49 mL) was added dropwise at 0 °C. The reaction mixture was stirred at the same temperature for 3 h. The solvent was removed at reduced pressure, stripping with toluene (2 × 2 mL) and Et_2O (2 × 2 mL) to remove the excess of trifluoroacetic acid. The residue was dissolved in CH_2Cl_2 (15 mL) and washed with saturated solution of $NaHCO_3$ (1 × 15 mL). The layers were separated and the aqueous layer was extracted with ethyl acetate (2 × 15 mL). The combined organic layers were dried with Na_2SO_4 and the solvent was removed in vacuo. Compound **10** (139,0 mg, 90 %) was obtained as a sticky solid. . ^{1}H NMR (300 MHz, $CDCl_3$): δ = 7.59 (s, 1H), 7.57-7.52 (m, 1H), 7.38-7.27 (m, 2H), 7.19-7.13 (m, 1H), 7.05-6.98 (m, 1H), 6.36-6.22 (m, 3H), 4.91 (s, 2H), 3.82 (s, 3H), 3.71 (s, 3H). ^{13}C NMR (75 MHz, $CDCl_3$): δ = 167.8, 159.6, 150.2, 147.7, 138.6, 133.7, 133.6, 130.3, 130.0, 126.7, 126.6, 109.4, 107.4, 104.1, 100.7, 71.7, 60.1, 50.8.

General procedure for the synthesis of compounds **12a-c.**
To a solution of the suitable acid **11a-c** (0.18 mmol) in abs. CH_2Cl_2 (1 mL) at 0 °C under N_2 atmosphere, EDC·HCl (0.19 mmol) and HOBt (0.19 mmol) were added. The reaction mixture was stirred at 0 °C for 1 h. After that, a solution of compound **10** (0.16 mmol) in abs. CH_2Cl_2 (0.7 mL) was added dropwise at 0 °C.

Then, DIPEA (0.32 mmol, 56 µl) was added dropwise at the same temperature and the reaction mixture was stirred at room temperature for 24 h. The reaction mixture was diluted with ethyl acetate (15 mL) and washed with a saturated solution of NH_4Cl (3 × 20 mL), saturated solution of $NaHCO_3$ (1 × 20 mL) and saturated solution of NaCl (1 × 20 mL). The organic layer was dried with Na_2SO_4 and the solvent was removed in vacuo The residue was purified by flash chromatography to give the corresponding compounds **12a-c**.

Methyl (E)-2-{2-[3-(2-methylbenzoylamino)-phenoxymethyl]-phenyl}-3-methoxy-acrylate **12a.**
Prepared according to general procedure from **10** and **11a.** Purified by flash chromatography (SiO_2, 5:1 hexane/acetone) (59.4 mg, 86 %). White solid. mp 79-82 °C. ^{1}H NMR (300 MHz, $CDCl_3$): δ = 7.59 (s, 1H), 7.58-7.54 (m, 2H), 7.52-7.45 (m, 1H), 7.39-7.30 (m, 3H), 7.30-7.20 (m, 4H), 7.20-7.13 (m, 2H), 6.73-6.66 (m, 1H), 4.98 (s, 2H), 3.83 (s, 3H), 3.64 (s, 3H), 2.49 (s, 3H). ^{13}C NMR (75 MHz, CDCl3): δ = 168.2, 168.1, 160.4, 159.5, 139.4, 136.7, 136.6, 136.2, 131.3, 131.1, 130.3, 129.9, 128.3, 127.7, 126.8, 126.0, 112.3, 111.5, 110.2, 106.5, 68.1, 62.2, 51.8. Anal. Calcd. for $C_{26}H_{25}NO_5$: C, 72.37; H, 5.84; N, 3.25. Found: C, 72.09; H, 5.86; N, 3.24.

Methyl (E)-2-{2-[3-(2-trifluoromethylbenzoylamino)-phenoxymethyl]-phenyl}-3-methoxyacrylate **12b.**
Prepared according to general procedure from **10** and **11b.** Purified by flash chromatography (SiO_2, 3:1 hexane/ethyl acetate) (62.2 mg, 80 %). Light grey solid. mp 62-66°C. ^{1}H NMR (300 MHz, $CDCl_3$): δ = 7.78-7.71 (m, 1H), 7.68-7.52 (m, 4H), 7.58 (s, 1H), 7.38-7.28 (m, 2H), 7.25-7.19 (m, 2H), 7.19-7.13 (m, 2H), 6.76-6.68 (m, 1H), 4.98 (s, 2H), 3.82 (s, 3H), 3.64 (s, 3H). ^{13}C NMR (100 MHz, CDCl3): δ = 168.2, 165.7, 160.4, 159.5, 138.8, 163.1, 132.3, 131.4, 131.1, 130.2, 129.9, 128.7, 128.3, 127.8, 126.6, 125.5, 121.9, 112.5, 111.8, 110.1, 106.8, 68.2, 62.2, 51.7. Anal. Calcd. for $C_{26}H_{22}F_3NO_5$: C, 64.33; H, 4.57; N, 2.89. Found: C, 64.57; H, 4.56; N, 2.89.

Methyl (E)-2-(2-{3-[(2-chloropyridine-3-carbonyl)-amino]-phenoxymethyl}-phenyl)-3-methoxyacrylate **12c.**
Prepared according to general procedure from **10** and **11c.** Purified by flash chromatography (SiO_2, 1:1 hexane/acetone) (47.3 mg, 65 %) as a white waxy solid. ^{1}H NMR (300 MHz, $CDCl_3$): δ = 8.50 (dd, *J* = 4.8, 1.9 Hz, 1H), 8.22-8.16 (m, 1H), 8.12 (dd, *J* = 7.6, 1.9 Hz, 1H), 7.59 (s, 1H), 7.58-7.52 (m, 2H), 7.42-7.30 (m, 2H), 7.30-7.20 (m, 2H), 7.20-7.13 (m, 1H), 6.77-6.70 (m, 1H), 4.99 (s, 1H), 3.84 (s, 3H), 3.67 (s, 3H). ^{13}C NMR (75 MHz, CDCl3): δ = 168.2, 165.2, 162.2, 154.8, 148.9, 147.2, 140.3, 139.2, 138.6, 133.7, 130.3, 129.7, 127.9, 128.0, 127.3, 127.2, 112.7, 110.0, 109.7, 105.9, 72.3, 54.0, 51.2. Anal. Calcd. for $C_{24}H_{21}ClN_2O_5$: C, 63.65; H, 4.67; N, 6.19. Found: C, 63.90; H, 4.65; N, 6.18.

3 References

1. Gutierrez, D. A., Lee, W.-C. C., Shen, Y. & Li, J. J. Palladium-catalyzed electrophilic C–H fluorination of arenes using oxazoline as a removable directing group. *Tetrahedron Lett.* **57,** 5372–5376 (2016).
2. Ye, X., Petersen, J. L. & Shi, X. Nickel-catalyzed directed sulfenylation of sp2 and sp3 C–H bonds. *Chem. Commun.* **51,** 7863–7866 (2015).
3. Kamal, A. *et al.* Synthesis and evaluation of N-((1-benzyl-1H-1,2,3-triazol-4-yl)methyl)nicotinamides as potential anticancer agents that inhibit tubulin polymerization. *Bioorg. Med. Chem.* **22,** 3465–3477 (2014).
4. Li, J., Qian, B. & Huang, H. Silver-catalyzed olefination of acetals and ketals with diazoesters to β-alkoxyacrylates. *Org. Lett.* **20,** 7090–7094 (2018).
5. Periyasamy, S. & Subbiah, S. Sodium iodide as a novel, chemoselective and highly efficient catalyst for N- tert-butoxy Carbonylation of amines at room temperature. *J. Chem. Pharm. Res.* **8,** 510–518 (2016).

4 Figures

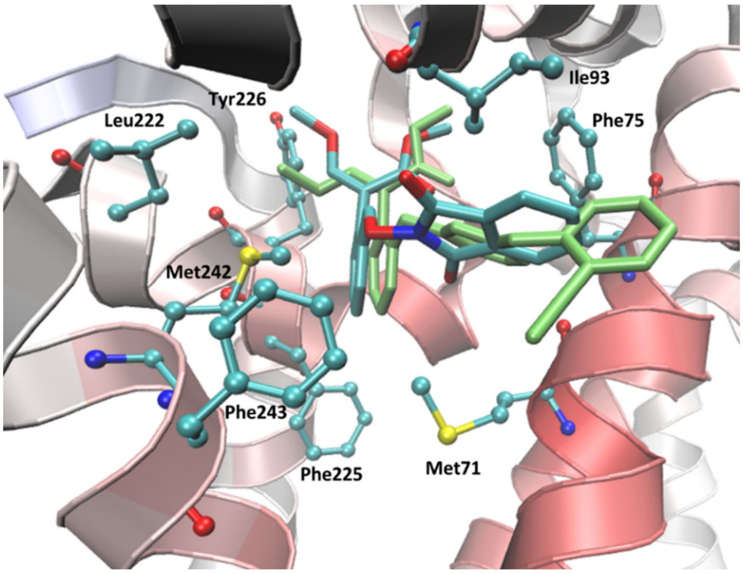

Figure S1. Comparison of the putative poses as computed for **5** and Axozystrobin (colored in light green) within the CytB binding site

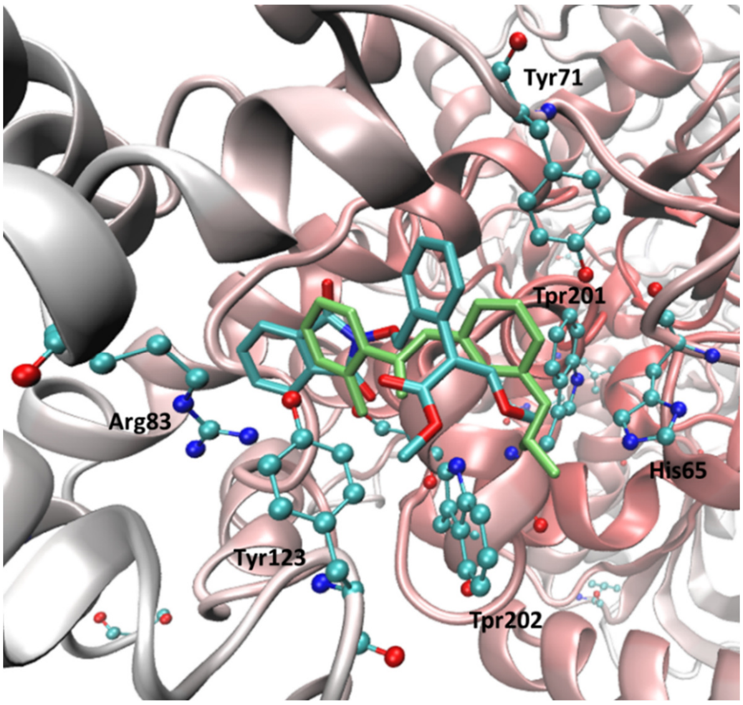

Figure S2. Comparison of the putative poses as computed for **5** and Mepronil (colored in light green) within the SDH binding site.
